# Neuregulin-1 attenuates mortality associated with experimental cerebral malaria

**DOI:** 10.1186/1742-2094-11-9

**Published:** 2014-01-17

**Authors:** Wesley Solomon, Nana O Wilson, Leonard Anderson, Sidney Pitts, John Patrickson, Mingli Liu, Byron D Ford, Jonathan K Stiles

**Affiliations:** 1 Department of Microbiology, Biochemistry and Immunology, Morehouse School of Medicine, Atlanta, GA, USA; 2 Cardiovascular Research Institute Morehouse School of Medicine, Atlanta, GA, USA; 3 Department of Pathology Morehouse School of Medicine, Atlanta, GA, USA; 4 Department of Neurobiology, Neuroscience Institute, Morehouse School of Medicine, Atlanta, GA, USA

**Keywords:** Neuregulin-1 (NRG-1), Pro-inflammatory, Anti-inflammatory, Blood–brain barrier (BBB), Inflammation, *Plasmodium berghei* ANKA (PbA), Adjunctive therapy, Malaria, Cerebral malaria (CM), Brain injury

## Abstract

**Background:**

Cerebral Malaria (CM) is a diffuse encephalopathy caused by *Plasmodium falciparum* infection. Despite availability of antimalarial drugs, CM-associated mortality remains high at approximately 30% and a subset of survivors develop neurological and cognitive disabilities. While antimalarials are effective at clearing *Plasmodium* parasites they do little to protect against CM pathophysiology and parasite-induced brain inflammation that leads to seizures, coma and long-term neurological sequelae in CM patients. Thus, there is urgent need to explore therapeutics that can reduce or prevent CM pathogenesis and associated brain inflammation to improve survival. Neuregulin-1 (NRG-1) is a neurotrophic growth factor shown to protect against brain injury associated with acute ischemic stroke (AIS) and neurotoxin exposure. However, this drug has not been tested against CM-associated brain injury. Since CM-associated brain injuries and AIS share similar pathophysiological features, we hypothesized that NRG-1 will reduce or prevent neuroinflammation and brain damage as well as improve survival in mice with late-stage experimental cerebral malaria (ECM).

**Methods:**

We tested the effects of NRG-1 on ECM-associated brain inflammation and mortality in *P. berghei* ANKA (PbA)-infected mice and compared to artemether (ARM) treatment; an antimalarial currently used in various combination therapies against malaria.

**Results:**

Treatment with ARM (25 mg/kg/day) effectively cleared parasites and reduced mortality in PbA-infected mice by 82%. Remarkably, NRG-1 therapy (1.25 ng/kg/day) significantly improved survival against ECM by 73% despite increase in parasite burden within NRG-1-treated mice. Additionally, NRG-1 therapy reduced systemic and brain pro-inflammatory factors TNFalpha, IL-6, IL-1alpha and CXCL10 and enhanced anti-inflammatory factors, IL-5 and IL-13 while decreasing leukocyte accumulation in brain microvessels.

**Conclusions:**

This study suggests that NRG-1 attenuates ECM-associated brain inflammation and injuries and may represent a novel supportive therapy for the management of CM.

## Background

Nearly 300 million persons each year are infected with *Plasmodium falciparum* (*P. falciparum*) infection, a subset of whom may develop severe anemia or a diffuse encephalopathy known as cerebral malaria (CM) [1]. CM accounts for 110,000 deaths annually in children and one in four survivors develop neurological complications (cortical blindness, epilepsy, and monoparesis) and cognitive disability (speech deficits, working memory, and executive function disability) [2-8]. Despite appropriate antimalarial treatment, mortality associated with CM may be as high as 30% in adults and 20% in children [7,9-11]. Thus, targeting parasite in acute disease is not sufficient to ameliorate persistent neurological sequelae and mortality associated with CM. Understanding immunopathogenic features such as brain inflammation and injury leading to fatal CM have led to the identification and development of small molecules or immunotherapeutics that may be used to stabilize the blood–brain barrier (BBB) and ameliorate CM-associated brain damage and mortality [12-14]. However, most of these interventions administered as prophylactics to prevent development of neurological signs failed to reverse CM-associated brain injuries or resulted in minimal therapeutic benefit, whereas others were deleterious [13,14]. The use of prophylactic strategies may not be clinically relevant as most patients who present to clinics have neurological abnormalities or clinical signs of CM. It is therefore important for new therapeutic strategies to ameliorate complications associated with late stages of CM to improve clinical outcomes while reducing risk of neurological sequelae in surviving CM patients. Clinical studies in human CM and murine experimental CM (ECM) indicate an exaggerated activation and dysregulation of host inflammatory processes including brain endothelial activation, and disruption of the BBB during the pathogenesis of the disease [15-18]. In fact, extensive research has linked strong host pro-inflammatory response to malaria disease states [19-21] and genetic studies have identified several immune regulatory and effector loci that possess mutations associated with susceptibility and resistance to human severe (cerebral) malaria [22-24]. Efforts underway to identify candidate therapeutics against CM have produced promising candidates including artovastatin, a statin with strong anti-inflammatory effects that effectively attenuates ECM [25-27]. Thus, interventions aimed at modulating the deleterious hyper-inflammatory response to malaria infection while protecting against brain damage will potentially bolster therapeutics against severe malaria.

Neuregulin-1 (NRG-1) is a member of the neuregulin family of growth factors that promotes survival and function of neuronal cells [28-31]. Studies have shown that NRG-1 attenuates tissue damage and immunopathology in animal models of acute brain injury (ABI) such as acute ischemic stroke (AIS), traumatic brain injury (TBI), and nerve agent poisoning [32-37]. There are clear pathophysiological similarities between CM and AIS, including an exaggerated host expression of pro-inflammatory factors that lead to increased vascular endothelial activation with upregulation of adhesion molecules, glial activation, focal inflammation, activation of apoptotic pathways and eventually brain damage and death [38-40]. Exogenous treatment with NRG-1 has been shown to significantly alter or inactivate inflammatory pathways associated with tissue damage during ischemic episodes [36]. Furthermore, NRG-1 reduces brain inflammation via inhibition of immune and oxidative stress mediators involved in the pathogenesis of focal ischemic brain damage [32]. Although NRG-1 has been studied extensively in AIS it has yet to be studied as a potential intervention against cerebral malaria. Using the *Plasmodium berghei* (*P. berghei*) ANKA (PbA) model of ECM, we tested the hypothesis that NRG-1 will reduce or prevent ECM-associated inflammation and improve survival in mice with late stage ECM. We show here that NRG-1 (1.25 ng/kg/day) significantly reduces ECM-associated brain and systemic inflammation and improves survival in mice with late-stage ECM.

## Methods

### Infection of mice with *P. berghei* ANKA

Six- to eight-week-old *C57BL/6 J* mice (Charles Rivers Laboratories, Wilmington, MA, USA) were housed in groups of four per cage on a 12 hr light/12 hr dark cycle with access to food *ad libitum* and water. Mice were allowed to acclimatize to their new environment for 3 days before experimentation. All experimental procedures were reviewed and approved by the Morehouse School of Medicine Institutional Animal Care and Use Committee (Permit Number 09–06). Procedures were performed with strict adherence to national regulations on animal care and experimentation with the use of Care of Laboratory Animal Resources (CLAR) guidelines to minimize pain. PbA was obtained from MR4, Manassas, VA, USA (BEI Resources Repository, NIAID, NIH; MR4 number MRA-311, deposited by TF McCutchan). Parasites were propagated in *C57BL/6 J* mice and a fresh blood sample from a passage mouse was used for each experiment. Experimental groups of mice were infected via intraperitoneal (i.p.) injection of 10^6^ PbA-infected red blood cells (pRBCs). Mice were sham-injected with 10^6^ non-infected red blood cells (RBCs).

### Clinical assessment of ECM

All animals were checked several times daily for mortality and ECM symptoms. For better estimation of the overall clinical status of mice during infection, simple behavioral tests (transfer arousal, locomotor activity, tail elevation, wire maneuver, contact righting reflex, and righting in arena) adapted from the **S**mithKline Beecham, **H**arwell, **I**mperial College, **R**oyal London Hospital, **p**henotype **a**ssessment (SHIRPA) protocol [41-43] were used. Infected mice display signs of ECM by day 5 or 6 post infection [41]. ECM is defined as the presentation of one or more signs of neurological deficit including ataxia, convulsions, limb paralysis, poor righting reflex, roll-over and coma [41]. Presentation of these signs were evaluated and scored to better assess severity of ECM in mice [44].

### Assessment of NRG-1 and artemether treatment in mice infected with or without PbA

Mice were selected and randomized into treatment groups after diagnosis with ECM on day 5 to 6 post infection. For survival experiments, 11 mice per group were used to obtain significant statistical data. To determine the therapeutic benefit of NRG-1 on ECM-associated brain damage and mortality and to compare NRG-1 with artemether (ARM) treatment, PbA-infected mice were treated daily via i.p. injection with 50-μl doses of NRG-1 (1.25 ng/kg/day, EGF-like domain, R & D Systems, Minneapolis, MN, USA) [NP_039250] or artemether prepared in coconut oil (25 mg/kg/day, Sigma-Aldrich, St Louis, MO, USA), from day 6 to day 9 post infection. PbA-infected mice treated daily with 50 μl saline solution (i.p.) from day 6 to day 9 post infection were used as the control. Mice were checked several times daily for mortality and signs of ECM neurological symptoms such as ataxia, loss of reflex and hemiplegia. All murine ECM experiments were terminated 19 days after PbA infection with animals euthanized accordingly. Parasite load was monitored periodically (beginning on day 5 post infection) by Giemsa staining of thin blood smears and assessed by counting the number of pRBCs per 1,000 erythrocytes.

### Assessment of leukocyte accumulation in brain parenchymal vessels during murine ECM in PbA-infected mice by H&E staining

To determine the effect of NRG-1 and ARM treatment on leukocyte accumulation in brain parenchymal vessels during murine ECM pathogenesis, PbA-infected *C57BL/6 J* mice were anesthetized with isoflurane inhalation and euthanized on day 5 and day 11 post infection. Mice were perfused with 10 ml of cold sterile phosphate-buffered saline to clear vessels of blood prior to collection of brain tissue (three mice per time point per treatment group). Whole brains were stored in formalin for fixation, embedded in paraffin, and sectioned at 10 μm. Sagittal sections of the brain (day 5 and day 11 post infection) were fixed in 4% paraformaldehyde and blocked with horse serum for 30 minutes at room temperature. Sections were stained with H&E and leukocytes in the blood vessels were quantified using an ocular grid calibrated with a × 400 magnification in an Axioskop 2 Plus microscope (Carl Zeiss Microscopy, Thornwood, NY, USA). The whole area of each section was similarly quantified with the ocular grid calibrated at × 40 magnification. Digital photos were captured by a high-resolution AxioCam HRc camera (Carl Zeiss Microscopy).

### Determination of the effect of NRG-1 on mRNA expression of factors involved in vascular endothelial activation and BBB integrity

To determine the effect of NRG-1 on mRNA expression of factors involved in vascular endothelial activation and BBB integrity, whole brain tissue from PbA-infected mice treated with either saline, ARM or NRG-1 (three mice per group per time point) was collected and homogenized in Trizol reagent (Life Technologies, Gaithersburg, MD, USA) and total RNA was extracted using RNeasy Mini Kit (Qiagen, Valencia, CA, USA). Briefly, chloroform (0.2 ml) was added to the homogenate, and the lysate mixed thoroughly. After centrifuging at 12,000 × g for 20 minutes at 4°C, the aqueous layer was transferred to a new tube. RNA was precipitated with 500 μl of isopropanol and pelleted by centrifuging at 12,000 × g for 20 minutes at 4°C. RNase-Free DNase Set (Qiagen) was used according to the manufacturer’s instructions to remove contaminating genomic DNA. DNase-treated RNA samples were subsequently stored at -80°C until ready to use. Reverse transcription of RNA samples was performed prior to quantitative PCR. cDNA was synthesized from up to 2 μg of total RNA iScript™ cDNA Synthesis Kit (Bio-Rad Laboratories, Hercules, CA, USA) using Multigene Gradient Thermal cycler (Labnet International, Inc. Edison, NJ, USA). The resulting cDNA was diluted 1:10 by addition of 180 μl of distilled water for quantitative PCR analysis. The primer sequences used for quantitative PCR are described in Table 1.

**Table 1:** Primer sequences used

**Target gene or mRNA**	**Primer 5′ - 3′**	
	**Forward**	**Reverse**
***HPRT***	GCTTTCCCTGGTTAAGCAGTACA	CAAACTTGTCTGGAATTTCAAATC
***ICAM-1***	GCCTCCGGACTTTCGATCTT	GTCAGGGGTGTCGAGCTTTG
***ANG-1***	ATGCTGTTCAAAACCACACG	TTTCAAGTCGGGATGTTTGAT
***ANG-2***	ATGTGGTGCAGAACCAGACA	GCAGCTCGAGTCTTGTCGTC
***C/EBPβ***	TCTACTACGAGCCCGACTGC	AGGTAGGGGCTGAAGTCGAT

The quantitative real-time PCR assay was performed using Bio-Rad C1000 thermal cycler (Bio-Rad Laboratories). Approximately 20 ng of cDNA was used in each 25 μl PCR reaction using the Bio-Rad iQ™ SYBR® Green Supermix (Bio-Rad Laboratories, Hercules, CA) and 50 μM of each primer. After a 15-minute incubation at 95°C, amplification was achieved by 39 cycles of a 15-s denaturation incubation at 95°C, followed by a 30-s annealing incubation at 55°C and 30-s extension incubation at 72°C. The identity and purity of the PCR product was confirmed by using dissociation curves and by checking the melting temperature of the PCR product, independently of the PCR reaction. To determine the relative amount of target cDNA present, the cycles to threshold (Ct) values of the target genes were compared with the basal expression of the housekeeping gene, hypoxanthine guanine phosphoribosyltransferase (*HPRT*). The average amount of *HPRT* present in each mouse group was used to normalized the quantity of target mRNA sequence against total RNA in each reaction. The differences in Ct values between *HPRT* and target gene of day 11 after infection of each group were compared with day 5 after infection-untreated control samples to determine the relative change in mRNA expression.

### Assessment of NRG-1 effects on expression of immune determinants of CM severity

To determine the effect of NRG-1 and ARM treatment on cytokine/chemokine levels, serum collected from blood harvested via cardiac puncture at pre-treatment (day 0 and day 5) and post treatment (day 11) from anesthetized mice (three to four mice per treatment group per day; pooled) was measured for levels of TNFα, IL-1α, IL-6, chemokine (C-X-C motif) ligand 10 (CXCL10), granulocyte colony stimulating factor (G-CSF), IL-5, and IL-13. Pooled serum samples were evaluated using Milliplex MAP mouse Cytokine/Chemokine bead-based immunoassay (Millipore, Billerica, MA, USA) coupled with the Luminex 200™ system (Austin, TX, USA) according to the manufacturer’s protocol. Samples were tested at a 1:2 dilution using optimal concentrations of standards and antibodies according to the manufacturer’s protocol.

### Statistical analysis

Results were expressed as means ± SD from at least three separate experiments performed in triplicate unless otherwise stated. Differences between means among the treatment groups were analyzed by using the Student *t*-test or one-way analysis of variance (ANOVA) with Holm-Sidak post-test methods where appropriate. Differences in survival among treatment groups were analyzed with Mantel-Cox log rank test. A *P*-value less than 0.05 was considered significant. Statistical analysis was performed with SigmaPlot (Version 10.0) with SigmaStat (Version 3.5) software for windows.

### Ethics statement

This study was carried out in strict accordance with the recommendations in the Guide for the Care and Use of Laboratory Animals of the National Institutes of Health. The Institutional Animal Care and Usage Committee (IACUC) of Morehouse School of Medicine (Permit Number 09–06) approved all protocols.

## Results

### NRG-1 therapy attenuates ECM-associated mortality

**Figure 1:**
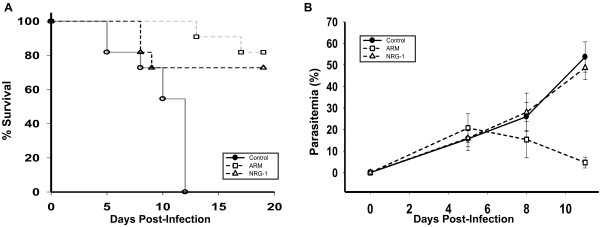
**Neuregulin-1 (NRG-1) therapy protects 73% of mice from fatal murine experimental cerebral malaria (ECM).***Plasmodium berghei* ANKA (PbA)-infected mice were treated intraperitoneally from day 6 to 9 post-infection with NRG-1 at 1.25 ng/kg/day (n = 11) and artemether (ARM) at 25 mg/kg/day (n = 11). **(A)** Survival improved after NRG-1 and ARM treatment compared to saline treatment (*P* <0.001, log rank test). **(B)** Parasite load was measured as the number of parasitized red blood cells (RBCs) in at least 1,000 RBC. The experiment is representative of three independent infections. Results shown are mean ± SD.

To test whether NRG-1 improves survival from ECM, PbA-infected *C57BL/6 J* mice were treated with recombinant human NRG-1 (1.25 ng/kg/day) or ARM (25 mg/kg/day). ECM-associated mortality was observed between days 5 and 12 post infection in PbA-infected mice sham-treated with saline, with mortality between 30% and 100% (Figure 1A). ARM treatment reduced mortality by 82% (*P* <0.001, Mantel-Cox, log rank) compared to saline treatment (Figure 1A). Mice treated with NRG-1 showed significantly reduced mortality at 73% (*P* <0.01, Mantel-Cox, log rank) compared to saline treatment (Figure 1A).

NRG-1 effect on parasite load was assessed before and after treatment. Parasite load in saline-treated mice increased markedly from day 5 to day 11 post infection by which time all the mice had been euthanized (Figure 1A and B). ARM treatment significantly reduced parasite load in PbA-infected mice as expected from 21% on day 5 post infection to <5% by day 11 post infection when compared with saline-treated mice on day 11 post infection, *P* <0.001 (Figure 1B). NRG-1-treated mice demonstrated improved survival despite no significant difference in parasite load compared to saline-treated mice (Figure 1A and B) suggesting that NRG-1 mediated attenuation of ECM was not via the reduction of parasite burden.

### NRG-1 treatment reduces leukocyte accumulation in brain microvasculature of PbA-infected mice

**Figure 2:**
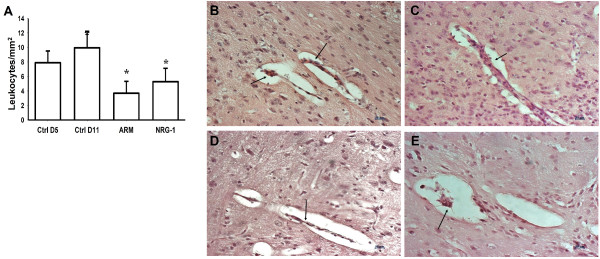
**Inhibition of leukocyte accumulation in the brain of *Plasmodium berghei *ANKA (PbA)**-**infected mice with experimental cerebral malaria (ECM) after treatment.** The number of intravascular leukocytes per mm^2^ of brain area was significantly decreased after neuregulin-1 (NRG-1) treatment **(A)**. Parenchymal vessels of untreated PbA-infected mice on day 5 **(B)**. Lumen of parenchymal vessels of saline-treated ECM mice on day 11 plugged with leukocytes (black arrows) **(C)**. Parenchymal vessels of **(D)** artemether (ARM)-treated mice and **(E)** NRG-1-treated mice showing remnants of adherent leukocytes after treatment (black arrows). Vascular congestion was observed in all saline-treated PbA-infected mice but was not seen in ARM or NRG-1 treated mice. Leukocyte counts are mean ± standard error. *P*-values less than 0.05 were considered significant. ^*^Statistical significance compared with control (Ctrl) day (D)5; ^§^statistical significance compared with Ctrl D11.

Marked leukocyte adherence and accumulation in brain vessels is linked to brain inflammation and is critical for murine ECM pathogenesis [41,45]. To determine the effect of NRG-1 therapy on brain inflammation in PbA-infected mice, the number of adherent leukocytes was quantified after treatment with NRG-1 on day 11 post infection. The numbers of leukocytes per vessel and per mm^2^ decreased after NRG-1 treatment when compared with saline treatment (Figure 2). Brain microvessels in mice treated with ARM showed significant reduction in leukocyte accumulation by day 11 post infection compared to saline-treated mice (Figure 2). Although NRG-1-treated mice had high peripheral parasitemia compared to ARM-treated mice, there were no significant differences in the accumulation of leukocytes in the brains of NRG-1-treated mice and ARM-treated mice (Figure 2).

### NRG-1 treatment decreases activation of brain vascular endothelium and promotes BBB stability in PbA-infected mice

Overproduction of pro-inflammatory factors promotes vascular endothelial activation and is deleterious to BBB integrity [46]. To investigate the effect of NRG-1 on activation of brain vascular endothelium and BBB integrity during ECM, mRNA levels of specific protein markers (angiopoietin-1 and -2, CCAAT enhancer-binding protein (C/EBP)β, and intercellular adhesion molecule-1 (ICAM-1)) that mediate endothelial activation [47-49] and BBB breakdown [50,51] were assessed.

**Figure 3:**
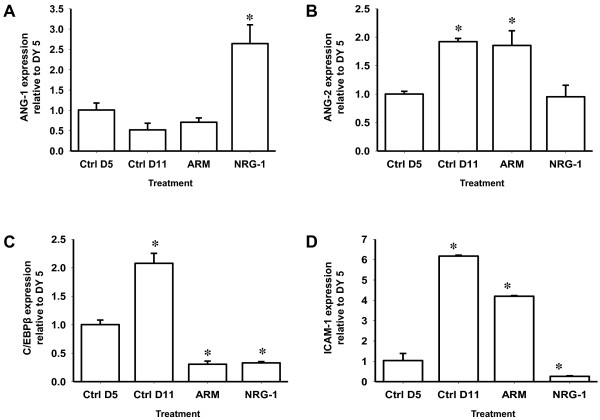
**Assessment of transcriptome associated with endothelium activation and blood–brain barrier integrity.** mRNA expression of angiopoietin-1 (ANG)-1 **(A)**, angiopoietin-2 **(B)**, CCAAT enhancer-binding protein (C/EBP)β **(C)**, and intercellular adhesion molecule-1 (ICAM-1) **(D)** in whole-brain homogenates of control (Ctrl), artemether (ARM), and neuregulin-1 (NRG-1)-treated *Plasmodium berghei*-infected mice (n = 5 per group). Expression measured by real-time PCR on day 11 post infection, is relative to the mean expression value in control mice day 5 post infection in brain samples and normalized with HPRT values. Treatment with NRG-1decreases endothelium activation and improves blood–brain barrier integrity. Columns and bars represent means ± standard error. Mean expression values were determined to be significantly different using one-way analysis of variance with the Holm-Sidak post-test method for all pairwise multiple comparison. A *P*-value <0.05 was considered significant. ^*^Statistically significant change compared with Ctrl day(D)5; ^§^statistically significant change compared with Ctrl D11. Note the different scales used in each graph.

Angiopoietin-1 and angiopoietin-2 are antagonistic regulators of endothelial cell activation and BBB function and integrity and are functional biomarkers that are used to predict fatal CM [52-54]. Expression of angiopoietin-1, a marker of vascular endothelial quiescence and BBB stability, increased in brain tissue of mice treated with NRG-1 compared with saline-treated mice, *P* <0.001 (Figure 3A). However, there was no significant difference in angiopoietin-1 levels between saline-treated and ARM-treated mice on day 11 when compared to day-5 untreated mice (Figure 3A). Expression of angiopoietin-2, a marker for BBB dysfunction, was significantly reduced in brain tissue of infected mice treated with NRG-1 compared to saline-treated mice, *P* <0.001 (Figure 3B). Conversely, expression of angiopoietin-2 increased significantly on day 11 in saline-treated and ARM-treated mice compared to day-5 untreated mice, *P* <0.001 (Figure 3B).

C/EBPβ is a critical regulator of acute-phase pro-inflammatory genes involved in host response to infections [55,56] and is implicated in the release of inflammatory and adhesion factors such as IL-6, TNFα, CD40, ICAM-1 and bioactive tachykinins responsible for neuroinflammation and tissue repair in the central nervous system [57-62]. C/EBPβ expression was significantly reduced in ARM-treated and NRG-1-treated mice compared to saline-treated mice, *P* <0.001 (Figure 3C). Expression of C/EBPβ increased significantly in the brains of saline-treated mice when compared to day 5 untreated mice, *P* <0.001 (Figure 3C). Expression of ICAM-1 which directly correlates with endothelial activation [47,63,64] was significantly reduced in brain of NRG-1 treated mice compared to saline-treated mice, *P* <0.001 (Figure 3D). ICAM-1 expression in NRG-1-treated mice was reduced to levels lower than that observed in day-5 untreated mice (Figure 3D). However, ICAM-1 expression increased significantly in saline-treated and ARM-treated mice compared to day-5 untreated controls, *P* <0.001 (Figure 3D).

### NRG-1 treatment modulates immune determinants of CM severity

**Figure 4:**
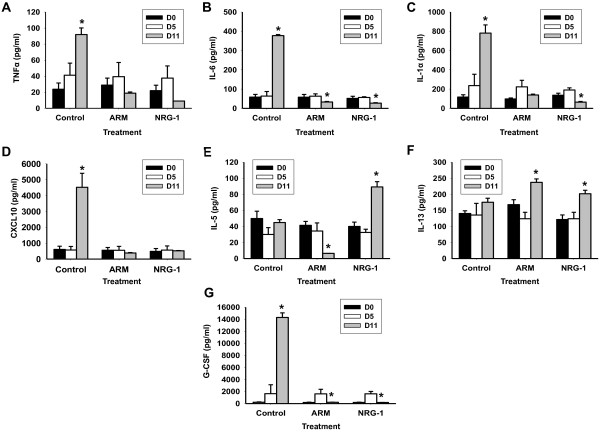
**Neuregulin-1 (NRG-1) inhibits expression of pro-inflammatory immune determinants of cerebral malaria (CM) severity.** Serum levels of pro-inflammatory, anti-angiogenic factors TNFα **(A)**, IL-6 **(B)**, IL-1α **(C)**, angiostatic factor CXCL10 **(D)**, anti-inflammatory factors IL-5 **(E)** and IL-13 **(F)**, and growth factor granulocyte colony stimulating factor, G-CSF **(G)**were measured before and after treatment. Serum samples were collected on day 5 and day 11 post infection (three to four mice per group). Results shown are mean ± SD. Statistical significance between means was determined with Student’s *t*-test; a *P*-value <0.05 was considered significant. ^*^Statistically significant change compared with control day **(D)**5; ^§^Statistically significant change compared with control D11. White bars indicate serum levels pre-treatment (day 5); gray bars indicate post-treatment levels (day 11). Note the different scales used in each graph.

Dysregulation of host pro-inflammatory factors plays a critical role in the pathogenesis of human CM and murine ECM. Previous studies showed overexpression of pro-inflammatory cytokines TNFα, IL-1α and IL-6 in CM patients promotes pathogenesis of CM (acute immune activation, promotion of adhesion molecules, leukocyte recruitment, fever and BBB disruption) and were associated with severe and lethal malaria [20,65-71]. We recently established that elevated levels of anti-angiogenic and apoptotic factor CXCL10 are associated with fatal CM in humans [72-74]. Moreover, CXCL10 plays a critical role in the development of murine ECM [75]. TNFα levels in saline-treated mice at day 11 increased significantly compared to untreated mice at day 5, *P* <0.05 (Figure 4A). However, TNFα serum levels were reduced in mice treated with NRG-1 and ARM compared to saline-treated mice, *P* <0.001 (Figure 4A). NRG-1 therapy significantly reduced serum IL-1α and IL-6 levels compared to saline-treated mice, *P* <0.001 (Figure 4B, C). Similarly, ARM treatment reduced serum levels of IL-1α and IL-6 compared to saline-treated mice, *P* <0.001 (Figure 4B, C). CXCL10 levels in saline-treated mice at day 11 increased significantly compared to untreated mice at day 5, *P* <0.001 (Figure 4D). Conversely, CXCL10 levels in mice treated with NRG-1 and ARM were significantly reduced at day 11 compared to saline-treated mice, *P* <0.001 (Figure 4D). In contrast, Th2 cytokines IL-5 and IL-13 associated with reduced severity of disease and increased protection against CM [76-79] were significantly elevated in serum after treatment with NRG-1compared to saline-treated mice, *P* <0.001 (Figure 4E, F). ARM therapy increased expression of IL-13 in PbA-infected mice although serum levels of IL-5 were markedly reduced after ARM treatment, *P* <0.001 (Figure 4E, F). Furthermore, increased levels of G-CSF, a neuronal growth factor, discriminate CM patients with poor disease outcome [20,80]. G-CSF levels increased significantly in saline-treated mice at day 11 compared to untreated mice at day 5, *P* <0.001 (Figure 4G). G-CSF levels were significantly reduced in infected mice treated with ARM and NRG-1 compared to saline-treated mice, *P* <0.001 (Figure 4G).

## Discussion

Despite prompt administration of optimal antimalarial treatment, mortality associated with CM remains unacceptably high, thus, prompting the development of adjunct therapeutics that can reduce or prevent CM pathology and associated mortalities [12,13] Recent studies have shown that NRG-1 was effective in treating ABI such as AIS and acute neurotoxin exposure by preventing neuroinflammation and neuronal tissue death [35,36], which are similar to those observed in CM. Furthermore, NRG-1 stabilizes the BBB and mediates inflammatory pathways to prevent tissue damage associated with brain injury [32,33,37]. Using a PbA ECM model that mimics significant features of human CM, we have demonstrated the effectiveness of NRG-1 therapy against ECM pathophysiology, and associated mortality.

Advances in drug therapies that eradicate malaria parasites are still unable to prevent mortality in up to 30% of CM patients. In humans, quinine and artemisinin derivatives (artesunate and artemether) are the mainstream drugs used to treat CM [81,82]. ARM was selected for use in this study as previous research has demonstrated that ARM was more effective against murine ECM than quinine, artemisinin and artesunate [41]. Despite anti-parasitic properties of ARM, mortality rates were as high as 18% in mice treated with ARM in the current study. However, no evidence of neurological dysfunction associated with ECM was observed in ARM-treated mice. This unacceptably high mortality in ARM-treated mice may be due to low efficacy of ARM against PbA that can lead to recrudescence and malarial anemia post treatment [41]. Furthermore, therapies targeting parasite eradication without addressing secondary effects of parasite infection, such as tissue damage and neurological complications, are inadequate for preventing mortalities. Thus, there is great need for adjunct therapeutics that target CM pathology that in conjunction with parasite-eradicating antimalarial agents can prevent mortality associated with CM.

Permanent or reversible neurological sequelae such as coma, residual epilepsy and cognitive deficits, are common clinical outcomes in CM patients. These neurological outcomes are associated with inflammatory cascades initiated by pathogen toxins that lead to widespread endothelial activation and brain damage (petechial hemorrhage and neuronal cell death) and involves inflammation-induced sequestration of infected RBCs [83-85]. Similarly, accumulation of leukocytes occurs in brain microvessels of PbA-infected mice that leads to vascular congestion and contributes to brain damage. [86,87]. Although parasitemia levels were high in PbA-infected mice treated with NRG-1, there was significant reduction in leukocyte accumulation in brain microvessels after NRG-1 treatment. This indicates that NRG-1 therapy effectively reduces brain inflammation associated with ECM pathogenesis even in the presence of high parasitemia.

Human CM and murine ECM are characterized by a dysregulated immune response leading to overexpression of pro-inflammatory cytokines including TNFα, IL-1α, IL-6, and CXCL10 [67-69,74,75]. These cytokines are secreted by T-cells, macrophages and endothelial cells in response to infection and play several roles that include promotion of acute immune response, leukocyte recruitment, BBB disruption and negative hypothalamic mediation during febrile illness [66,70,88-93]. Plasma and cerebrospinal fluid levels of TNFα, IL-1α and IL-6 are increased in children with CM [20,68] suggesting their role in human CM. We previously reported that increased plasma and cerebrospinal fluid levels of CXCL10 predict fatal CM [72-74] and mice deficient in the *CXCL10* gene are partially protected against murine ECM [75]. NRG-1 therapy significantly reduced serum TNFα, IL-1α, IL-6, and CXCL10 levels while improving survival against ECM. High serum levels of the growth factor G-CSF have been shown to correlate with fatal CM in humans [80]. However, NRG-1 reduced G-CSF, suggesting amelioration of pathogenic pathways that leads to induction of G-CSF observed in fatal CM [20,80]. Thus, further study is warranted to determine the role of G-CSF in severe disease and the NRG-1 protective effect in reducing G-CSF production. Furthermore, there is growing evidence of the role for anti-inflammatory factors, IL-5 and IL-13 in protection against malarial disease. In a population of south Asian malaria patients, increased levels of IL-5 was associated with reduced severity of disease [79]. Genetic studies in African and south-east Asian populations have linked IL-13 to protection against cerebral malaria and show that polymorphisms that alter IL-13 production may increase risk of severe malaria [76-78]. In the present study, NRG-1 enhanced production of IL-5 and IL-13, and suggests NRG-1 promotes anti-inflammatory factors while dampening pro-inflammatory factors to ameliorate CM pathogenesis.

Angiopoietin-1 (a biomarker of endothelium quiescience and stability) and vascular permeability factor angiopoietin-2 (marker of vascular barrier breakdown) are potent modulators of vascular inflammation, endothelial activation and BBB function [49,94-96]. Angiopoietin-1 stabilizes the vascular endothelium barrier [97] and regulates the activity of BBB permeability factors such as platelet-activating factor (PAF), vascular epithelial growth factor (VEGF), ELAM-1, bradykinin, thrombin and histamine [98-101]. Increased activity of angiopoietin-1 promotes endothelial survival [102,103], modulates plasma leakage [100,104,105] and reduces vascular inflammation by inhibiting ICAM-1, vascular cell adhesion molecule-1 (VCAM-1) and E-selectin expression [106]. Conversely, pro-inflammatory cytokines TNF-α, IL-1β and vascular permeability factor, VEGF, mediate the release of angiopoietin-2, an antagonist to angiopoietin-1 [48,107], that promotes increased vascular inflammation [108], disruption of angiogenesis [109], endothelial cell death [110] and vascular regression [110,111]. Moreover, angiopoietin-2 expression is elevated in response to endothelial activation, hypoxia and ischemia [107,112-114]. In human CM, high levels of angiopoietin-2 and low levels of angiopoietin-1 are linked to CM severity and studies suggest these angiogenic factors function as prognostic biomarkers that can discriminate severe CM from mild malaria and predict fatal CM outcome [53,54]. Nakaoka *et al*. show that NRG-1 stimulates expression of angiopoietin-1 [115] and increased expression of angiopoietin-1 inhibits release or activity of angiopoietin-2 [107,116]. In the present study, NRG-1 treatment increased expression of angiopoietin-1, thus promoting endothelial barrier function and integrity during ECM, while modulating angiopoietin-2 expression in the brains of PbA-infected mice.

Parasite sequestration and activation of endothelial cells by infected erythrocytes and pro-inflammatory cytokines are hallmark events in the brain pathology of pediatric CM patients [64,117,118]. Parasite sequestration and endothelial activation correlate with an increase in adhesion molecules such as ICAM-1 and VCAM-1 that bind infected erythrocytes, influence leukocyte migration and promote further release of pro-inflammatory cytokines [119-121]. ICAM-1 is a marker of endothelial activation whose expression is upregulated on the vascular endothelium in the brain in murine and human CM [64,122-124]. ICAM expression is induced by pro-inflammatory cytokines such as TNF-α, IFN-γ and VEGF [47,125,126]. In human CM, increased ICAM-1 levels are associated with disease severity [63,119,127]. Furthermore, murine ECM studies show increased expression of ICAM-1 contributes to the development of ECM [47,128,129]. Previous studies indicate NRG-1 reduces the expression of ICAM-1 following ischemic stroke [32]. NRG-1 increases activity of PI3-kinase [130,131] which suppresses VEGF-mediated expression of ICAM-1 on endothelial cells [106,126]. Additionally, C/EBPβ is a critical regulator of acute host-response to infections and neuroinflammation [55-58,60] that stimulates release of inflammatory and adhesion factors such as IL-6, TNFα, CD40 and ICAM-1, thus contributing to ECM development [56-59,61]. In this study, NRG-1 treatment of murine ECM demonstrated inhibition of ICAM-1 and C/EBPβ in the ECM brain while reducing leukocyte adhesiveness and accumulation in brain microvessels.

NRG-1 was recently used as a treatment for heart failure and showed significant efficacy for improving cardiac function in a phase-II patient study [132-134]. In this study, patients received placebo or NRG-1 at a dose of 0.3 to 1.2 μg/kg/day intravenously for 10 days, in addition to standard drug therapies. During a follow-up period 11 to 90 days after study initiation, NRG-1 significantly improved heart function in patients and the effective doses were shown to be safe and tolerable. Two additional clinical trials to determine the ability of NRG-1 to improve cardiac function after heart failure have been initiated in the US (ClinicalTrails.gov identifiers NCT01258387 and NCT01541202). During the period of study, no severe events were observed in either healthy or impaired patients.

## Conclusions

The use of recombinant human NRG-1 against acute brain injury is being tested in experimental models [32,34-36,135,136]. Recent and ongoing clinical trials provide evidence indicating efficacy and safety of recombinant human NRG-1 against chronic heart failure and vascular remodeling [132-134]. The efficacy of NRG-1 treatment against murine ECM provides compelling evidence for developing NRG-1 and NRG-like drugs for the treatment and management of CM patients. By inhibiting systemic and brain inflammation resulting from ECM pathogenesis, NRG-1 therapy improved survival in mice with late-stage ECM. The ability of NRG-1 to affect a range of functionally related CM inflammatory mediators increases the likelihood that such an effect will translate to human CM to protect against human CM pathologies. We propose further investigation of NRG-1 as a supportive therapy alongside current antimalarial agents in the management of CM.

## Abbreviations

ABI: Acute brain injury; AIS: Acute ischemic stroke; ANOVA: Analysis of variance; ARM: Artemether; BBB: Blood–brain barrier; C/EBP: CCAAT enhancer-binding protein; CM: Cerebral malaria; Ct: Cycles to threshold; CXCL: Chemokine (C-X-C motif) ligand; ECM: Experimental cerebral malaria; G-CSF: Granulocyte colony stimulating factor; H&E: Hematoxylin and eosin; ICAM: Intercellular adhesion molecule-1; IL: Interleukin; i.p.: Intraperitoneal; NRG-1: Neuregulin-1; PbA: *Plasmodium berghei* ANKA; pRBC: PbA-infected red blood cell; RBC: Red blood cell; TBI: Traumatic brain injury; TNF: Tumor necrosis factor; VEGF: Vascular epithelial growth factor; VCAM-1: Vascular cell adhesion molecule-1.

## Competing interests

The authors declare that they have no competing interests.

## Authors’ contributions

WS designed and performed experiments, analyzed data and wrote the paper. NW performed experiments, analyzed data and wrote the paper. LA designed and performed experiments. SP designed and performed experiments. JP designed experiments and analyzed data. ML designed and performed experiments and analyzed data. BF designed experiments, analyzed data and assisted in the drafting this manuscript. JS conceived the study, designed and supervised experiments and assisted in the drafting this manuscript. All authors read and approved the final manuscript.
